# Changes in Left Ventricular Ejection Fraction after Mitral Valve Repair for Primary Mitral Regurgitation

**DOI:** 10.3390/jcm10132830

**Published:** 2021-06-26

**Authors:** Kyoung-Woon Joung, Seon-Ok Kim, Jae-Sik Nam, Young-Jin Moon, Hyeun-Joon Bae, Ji-Hyun Chin, Sung-Ho Jung, In-Cheol Choi

**Affiliations:** 1 Department of Anesthesiology and Pain Medicine, Asan Medical Center, University of Ulsan College of Medicine, Seoul 05505, Korea; baram0403@naver.com (K.-W.J.); jaesik_nam@naver.com (J.-S.N.); yjmoon@amc.seoul.kr (Y.-J.M.); joshpheonix1@gmail.com (H.-J.B.); icchoi@amc.seoul.kr (I.-C.C.); 2 Department of Clinical Epidemiology and Biostatistics, Asan Medical Center, Seoul 05505, Korea; seonok@amc.seoul.kr; 3 Department of Thoracic and Cardiovascular Surgery, Asan Medical Center, University of Ulsan College of Medicine, Seoul 05505, Korea; csjung@amc.seoul.kr

**Keywords:** left ventricular end-systolic diameter, left ventricular ejection fraction, mitral valve repair, chronic primary mitral regurgitation

## Abstract

This study sought to identify the short- and long-term changes in left ventricular ejection fraction (LVEF) after mitral valve repair (MVr) in patients with chronic primary mitral regurgitation according to preoperative LVEF (pre-LVEF) and preoperative left ventricular end-systolic diameter (pre-LVESD). This study evaluated 461 patients. Restricted cubic spline regression models were constructed to demonstrate the long-term changes in postoperative LVEF (post-LVEF). The patients were divided into four groups according to pre-LVEF (<50%, 50–60%, 60–70%, and ≥70%). The higher the pre-LVEF was, the greater was the decrease in LVEF immediately after MVr. In the same pre-LVEF range, immediate post-LVEF was lower in patients with pre-LVESD ≥ 40 mm than in those with pre-LVESD < 40 mm. The patterns of long-term changes in post-LVEF differed according to pre-LVEF (*p* for interaction < 0.001). The long-term post-LVEF reached a plateau of approximately 60% when the pre-LVEF was ≥50%, but it seemed to show a downward trend after reaching a peak at approximately 3–4 years after MVr when the pre-LVEF was ≥70%. The patterns of short- and long-term changes in post-LVEF differed according to pre-LVEF and pre-LVESD values in patients with chronic primary mitral regurgitation after MVr.

## 1. Introduction

Mitral valve surgery is recommended for chronic primary mitral regurgitation (MR) depending on the patient’s symptoms and left ventricular (LV) systolic function according to the 2017 American College of Cardiology/American Heart Association (ACC/AHA) guidelines [1]. LV systolic function is an important factor in determining the timing of surgery, and early intervention can be performed before the onset of LV systolic dysfunction [1]. LV ejection fraction (LVEF) and LV end-systolic diameter (LVESD) are used to assess the LV systolic dysfunction in patients with chronic MR [1].

Some studies have investigated the changes in LVEF after mitral valve surgery in patients with chronic severe MR [2,3,4,5,6,7]. In a previous study that included patients with severe degenerative MR with normal preoperative LVEF (pre-LVEF), LV dysfunction was observed in 18% of the patients immediately after mitral valve repair (MVr) and the LV dysfunction recovered in only two thirds of the patients during long-term follow-up [7]. Another study investigated the changes in postoperative LVEF (post-LVEF) at several time points, and observed an early decrease followed by a gradual increase for up to 5 years after surgery [4]. However, it may be difficult to conclude how LVEF serially changes postoperatively based only on these studies. Because the measurement time points were arbitrarily divided into distinct groups, the detailed changes within the same time interval could have been neglected in previous studies [4,7].

No study to date has examined the serial long-term changes in LVEF after MVr in patients with chronic primary MR with a wide range of pre-LVEF values. Identifying such changes may be helpful in predicting long-term outcomes after MVr in these patients based on pre-LVEF values. In addition, the extent of the decrease in LVEF immediately after MVr has not been extensively evaluated in patients with chronic MR, although a decrease in LVEF has been known to occur immediately after MVr in patients with chronic MR [4,7]. In the current study, we aimed to investigate the short- and long-term changes in LVEF after MVr in patients with chronic primary MR with a wide range of pre-LVEF values.

## 2. Materials and Methods

### 2.1. Study Population

This retrospective observational study included patients who underwent MVr for chronic MR with grade ≥ moderate caused by excessive motion of mitral valve leaflets, at Asan Medical Center (Seoul, Korea) from January 2005 to July 2015. Patients were excluded if remnant MR with grade ≥ moderate (moderate was defined as proximal isovelocity surface area radius ≥4 mm and <8 mm at the aliasing velocity of 40 cm/s) was detected at the immediate postoperative echocardiography; if they had coronary artery disease, concomitant aortic valve disease of grade ≥ mild, infective endocarditis, rheumatic MR, and acute MR; if they underwent redo surgery, concomitant coronary artery bypass graft surgery, or repeated mitral valve surgery after the index hospitalization; and if they did not have at least one postoperative echocardiographic examination. The research protocol was approved by our Institutional Review Board (AMC IRB 2017-0052), which waived the requirement for written informed consent. Data were acquired from a retrospective review of electronic medical records.

### 2.2. Echocardiographic Data

All patients underwent transthoracic echocardiographic examination before and during the follow-up after MVr. The details of the echocardiographic examination, including the assessment of MR severity, are described in the supplementary methods. Our institution followed the standards and techniques recommended by the American Society of Echocardiography for measuring MR severity [8,9].

Preoperative echocardiographic data obtained closest to the day of surgery were used in the analysis. At our institution, all patients undergo routine echocardiographic evaluation before discharge. The details of the analyzed immediate postoperative echocardiographic data are described in the supplementary methods.

The measurements for LVEF, LV end-systolic and LV end-diastolic volume index (LVESVI, LVEDVI), left atrial (LA) diameter, ratio of peak early diastolic velocity of mitral inflow to mitral annulus early diastolic velocity (E/e’), and the pressure gradient calculated from peak tricuspid regurgitation (PG_TR_) were collected.

In addition, we calculated the midwall fractional shortening (mFS) to assess LV contractility. The details of the calculation of actual and predicted mFS, circumferential end-systolic wall stress (cESS), and stress corrected mFS (sc-mFS) are described in the supplementary methods.

During the follow-up, re-developed MR was defined as the recurrence of MR with grade ≥ moderate, regardless of the measurement time point. All-cause mortality was obtained during the follow-up.

### 2.3. Statistical Analysis

Continuous data are presented as mean ± standard deviation or median (interquartile range), and categorical data are presented as frequencies (percentages). The patients were classified into four groups according to pre-LVEF: Gr_<50_ = pre-LVEF < 50%, Gr_50–60_ = 50% ≤ pre-LVEF < 60%, Gr_60–70_ = 60% ≤ pre-LVEF < 70%, and Gr_≥70_ = pre-LVEF ≥ 70%. We adopted a cut-off of 40 mm for preoperative LVESD (pre-LVESD) to indicate LV dysfunction, as suggested in the ACC/AHA guidelines for patients with chronic primary MR [1].

The P for trend test using linear regression analysis or Spearman’s correlation analysis was performed to investigate whether the immediate postoperative echocardiographic parameters had a linear trend across the four groups. To evaluate the effect of time on post-LVEF, linear mixed models were constructed, with group, time, and the interaction between group and time as fixed effects, and patient effect as random effects. Kaplan-Meier analysis was performed to compare long-term mortality using log-rank sum test, and Bonferroni correction was used as the post hoc test (adjusted α = 0.05/6 = 0.0083). Multivariable Cox proportional hazards regression analysis was performed to evaluate the impact of pre-LVEF groups on long-term mortality. *p* < 0.05 was considered statistically significant. The other details are described in the supplementary methods.

## 3. Results

**Figure 1:**
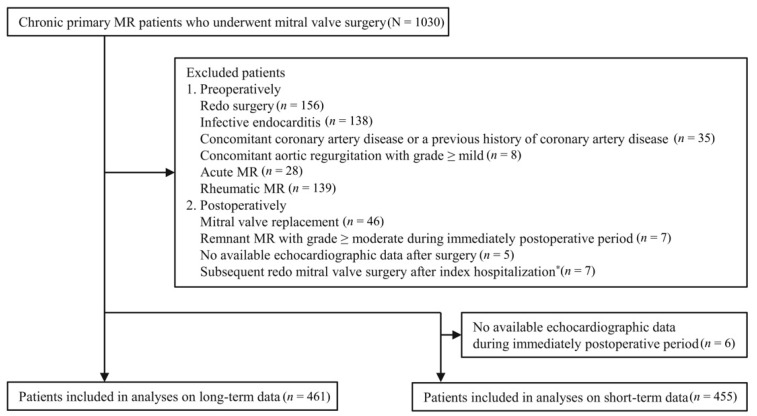
Flow diagram. MR, mitral regurgitation. * Included 1 patient with multiple echogenic masses in the mitral valve, 3 with severe MR development, 2 with significant MR development with hemolytic anemia, and 1 with infective endocarditis.

During the study period, 1030 patients underwent mitral valve surgery for MR. Among them, 569 did not satisfy the inclusion criteria (Figure 1); thus, the remaining 461 patients were evaluated. No patients underwent heart transplantation or coronary artery intervention during the follow-up. There were 49 patients who were diagnosed with heart failure.

A total of 2654 echocardiographic examinations were performed during 3.6 (1.8–7.1) years of follow-up. The immediate postoperative echocardiography, performed at 4.0 (3.0–5.0) days after the surgery, was analyzed in 455 patients (Figure 1).

The patients’ demographic, clinical, and preoperative echocardiographic data are presented in Table 1. Gr_<50_, Gr_50–60_, Gr_60–70_, and Gr_≥70_ comprised 15, 76, 284, and 86 patients, respectively. The proximal isovelocity surface area radius was 13.2 ± 4.0, 13.9 ± 4.0, 13.6 ± 3.2, and 14.3 ± 3.4 mm in Gr_<50_, Gr_50–60_, Gr_60–70_, and Gr_≥70_, respectively (*p* = 0.431). The comparisons of preoperative PG_TR,_ E/e’, and LA across groups are described in the supplemental results (Supplementary Figure S1).

The immediate postoperative LVEDVI (post-LVEDVI) decreased more in the order of ascending pre-LVEF groups (*P* for trend < 0.001) (Table 2). The amount of change between preoperative LVESVI (pre-LVESVI) and immediate postoperative LVESVI (post-LVESVI) tended to increase from negative to positive values in the order of ascending pre-LVEF groups (*P* for trend < 0.001) (Table 2).

**Figure 2:**
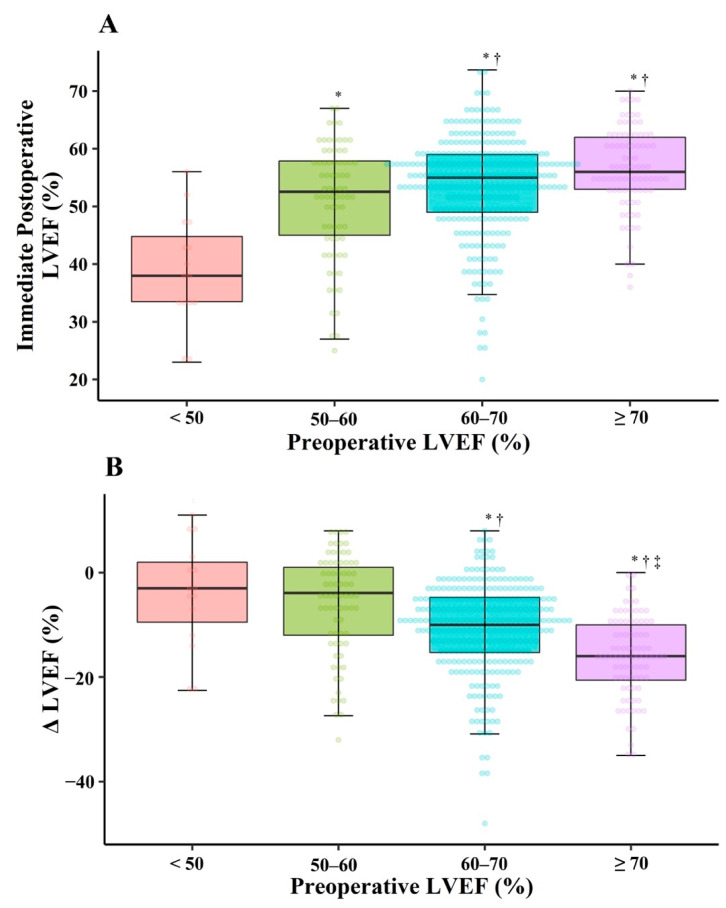
Immediate postoperative LVEF (**A**) and Δ LVEF (**B**) after mitral valve repair for chronic primary mitral regurgitation according to preoperative LVEF. LVEF: left ventricular ejection fraction; Δ LVEF: immediate postoperative LVEF minus preoperative LVEF. * *p* < 0.05 versus pre-LVEF < 50%, ^†^ *p* < 0.05 versus pre-LVEF 50–60%, ^‡^ *p* < 0.05 versus pre-LVEF 60–70%.

**Figure 3:**
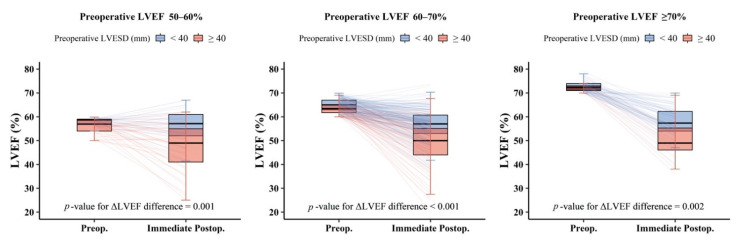
Immediate postoperative LVEF after mitral valve repair for chronic primary mitral regurgitation according to preoperative LVESD. LVEF: left ventricular ejection fraction; LVESD: left ventricular end-systolic diameter.

The immediate post-LVEF values were 39.0 ± 9.4%, 50.9 ± 9.9%, 53.8 ± 8.6%, and 56.5 ± 7.2% in Gr_<50_, Gr_50–60_, Gr_60–70_, and Gr_≥70_, respectively (Figure 2A). The higher the pre-LVEF was, the greater was the decrease in immediate post-LVEF (*P* for trend <0.001) (Figure 2B). When considering pre-LVESD ≥ 40 and < 40 mm separately in the same pre-LVEF range, the immediate post-LVEF decreased more in patients with pre-LVESD ≥ 40 mm than in those with pre-LVESD < 40 mm (each *p* < 0.001) (Figure 3). We could not perform this comparison in Gr_<50_, because there was no patient with pre-LVESD < 40 mm in this group. The result about immediate post-LVEF in patients excluding those with preoperative atrial fibrillation (n = 81) are described in the Supplementary Table S1.

**Figure 4:**
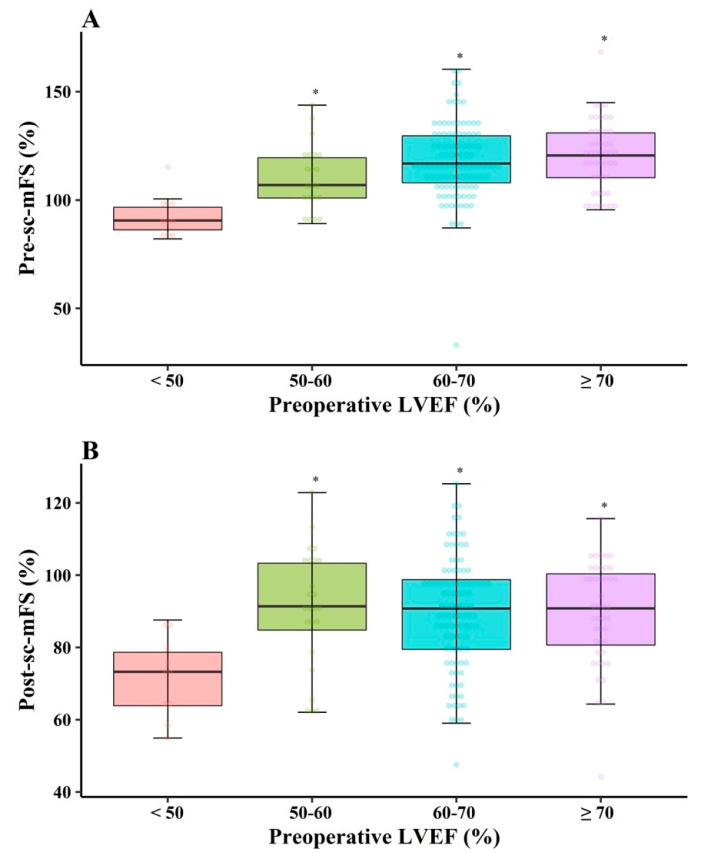
Preoperative (**A**) and immediate postoperative (**B**) sc-mFS after mitral valve repair for chronic primary mitral regurgitation. sc-mFS: stress-corrected midwall fractional shortening; LVEF: left ventricular ejection fraction. * *p* < 0.00833 versus pre-LVEF < 50%.

The preoperative sc-mFS (pre-sc-mFS) values were >100%, except in Gr_<50_ (Figure 4A). Conversely, the immediate postoperative sc-mFS (post-sc-mFS) values were <100% in all groups, and were similar in the comparison of pairs among Gr_50–60_, Gr_60–70_, and Gr_≥70_ (Figure 4B).

**Figure 5:**
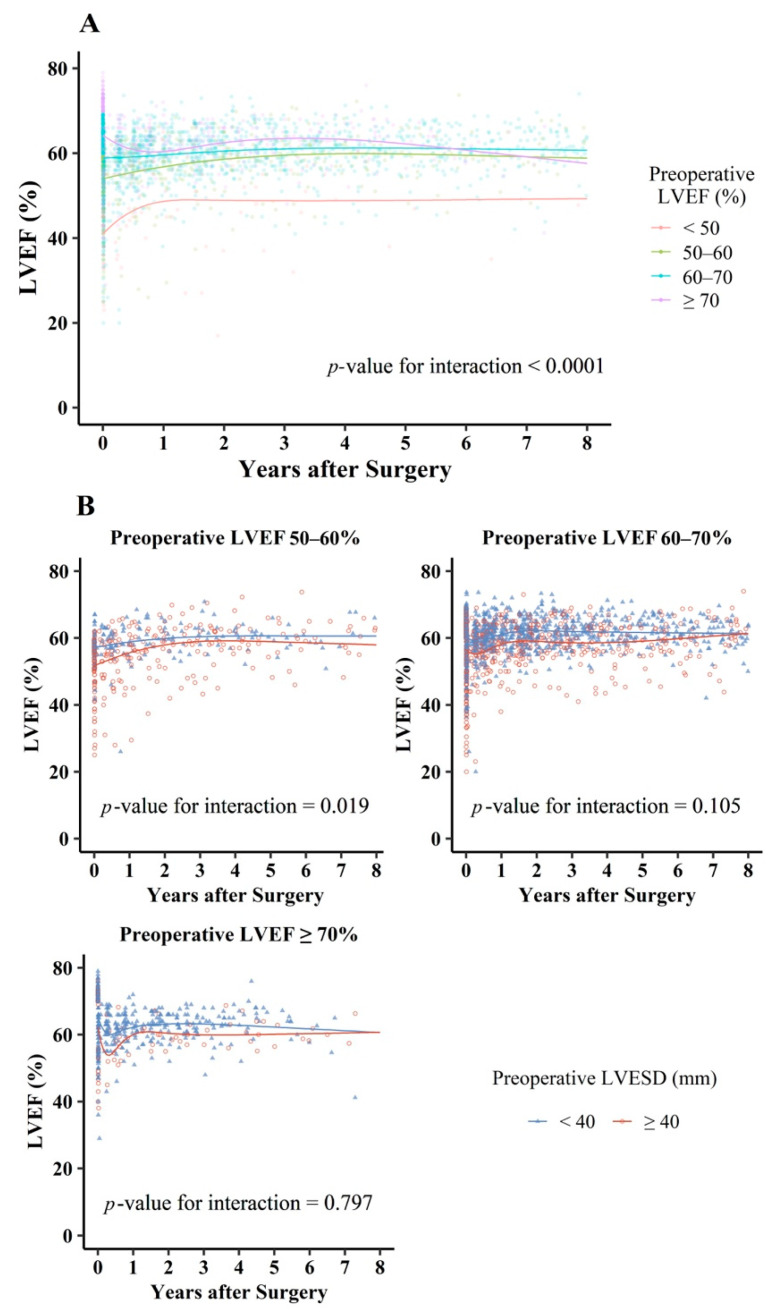
Restricted cubic spline curves of LVEF changes after mitral valve repair for chronic primary mitral regurgitation according to preoperative LVEF (**A**) and preoperative LVESD (**B**). LVEF: left ventricular ejection fraction; LVESD: left ventricular end-systolic diameter.

With respect to long-term follow-up, the patterns of changes in post-LVEF differed according to pre-LVEF (Figure 5A). In Gr_<50,_ post-LVEF increased until about 1 year after MVr, and thereafter showed a plateau of approximately 50%_._ In Gr_50–60_ and Gr_60–70_, post-LVEF increased until about 3–4 years after MVr, and thereafter formed a plateau of approximately 60% in the long-term. Conversely, in Gr_≥70_, post-LVEF decreased and thereafter subsequently increased until approximately 3 years, after which it seemed to decrease to a lower level than that in Gr_50–60_ over time. After excluding patients with re-developed MR (n = 59), similar patterns were observed in the long-term changes in post-LVEF (Supplementary Figure S2). Furthermore, we constructed separate models according to pre-LVESD (≥40 versus <40 mm) in the same pre-LVEF range (Figure 5B). Across all groups, for long-term changes in post-LVEF, higher post-LVEF was observed in patients with pre-LVESD < 40 mm than in those with pre-LVESD ≥ 40 mm.

**Figure 6:**
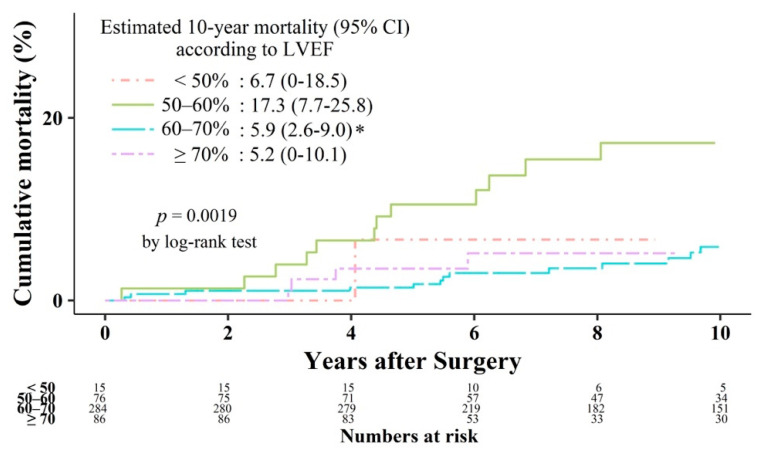
Kaplan–Meier curves of mortality. LVEF: left ventricular ejection fraction.

A total of 33 patients died during the follow-up. Kaplan–Meier analysis showed a difference in mortality rate among the groups (*p* = 0.0019), with the lowest mortality rate in Gr_60–70_ (Figure 6). After adjustment for age, sex, and Charlson comorbidity index, the morality rate was lower in Gr_60–70_ than in Gr_50–60_. The hazard ratio of mortality in Gr_50–60_ was 3.82 (95% confidence interval, 1.77–8.27; *p* = 0.001), when Gr_60–70_ was considered as a reference group.

## 4. Discussion

The current study found that the short- and long-term changes in LVEF after MVr differed according to pre-LVEF and pre-LVESD values in patients with chronic primary MR. The principal findings were as follows: (1) the higher the pre-LVEF was, the greater was the decrease in LVEF immediately after MVr; (2) the long-term post-LVEF reached a plateau of approximately 60% when the pre-LVEF was ≥50%, but seemed to show a downward trend after reaching a peak at approximately 3–4 years after MVr when the pre-LVEF was ≥70%; (3) among patients with the same pre-LVEF, the post-LVEF was lower in those with pre-LVESD ≥ 40 mm than in those with pre-LVESD < 40 mm during both short- and long-term follow-ups; (4) the long-term mortality rate was lowest in patients with 60% ≤ pre-LVEF < 70%.

Theoretically, the decrease in LVEF immediately after MVr can be attributed to a decrease in preload, an increase in afterload, or a decrease in contractility of left ventricle. When exploring Gr_60–70_ and Gr_≥70,_ or the patient groups with a LVEF considered normal for chronic primary MR according to the 2017 ACC/AHA guidelines [1], our results showed that post-LVEDVI decreased more in Gr_≥70_ than in Gr_60–70_. This might have led to a greater decrease in post-LVEF in Gr_≥70_ than in Gr_60–70_. We found that immediate postoperative cESS decreased more in Gr_≥70_ than in Gr_60–70_ (Table 2); therefore, it theoretically makes sense for immediate post-LVESVI to decrease more in Gr_≥70_ than in Gr_60–70_, because LVESVI is mainly dependent on afterload. However, our results showed that immediate post-LVESVI did not change in Gr_≥70_ but that it decreased in Gr_60–70_. From these findings, we could not exclude the effect of intrinsic LV contractility, which could be revealed in the absence of volume overload, on the immediate post-LVEF.

In this study, we assessed sc-mFS which provided an afterload-independent estimate of LV systolic function [10]. Because motion at the endocardial surface is greater than that predicted by sarcomere shortening alone as a result of cross-fiber shortening, LVEF does not necessarily reflect myocardial contractility [11]. To overcome this drawback, mFS was compared in our study. It is notable that the immediate post-sc-mFS values were <100% in all groups, indicating that LV systolic function might have been impaired during the immediate postoperative period. Myocardial stunning, which occurs after cardiopulmonary bypass, may be a reason for this observation. However, myocardial stunning typically resolves over 48–72 h after ischemia [12]. Considering that immediate postoperative echocardiographic examination was performed at 4.0 (3.0–5.0) days after surgery, we inferred that immediate post-LVEF and immediate post-sc-mFS may represent preoperative intrinsic LV contractility that was masked by a compensation mechanism for LV volume overload, rather than myocardial stunning.

Circumferential fiber contraction has been reported to aid in maintaining global ventricular systolic function in patients with severely impaired longitudinal fiber function, so that LVEF can be preserved [13,14,15]. We showed that the pre-sc-mFS increased with an increase in pre-LVEF and that most of the pre-sc-mFS values were >100%. These results may imply that the pre-LVEF increased with greater activation of the compensation mechanism for LV volume overload. Patients with supra-normal LVEF may potentially have more severe MR and hence substantially reduced afterload, resulting in a higher LVEF. Indeed, our results showed that cESS in Gr_≥70_ was lower than that in Gr_60–70_, although there was no statistically significant difference. Furthermore, preoperative PG_TR_ was higher in Gr_≥70_ than in Gr_60–70_, with similar E/e’ and LA diameter, showing that all values exceeded the normal ranges (Supplementary Figure S1). These findings implied a similarly increased LA pressure but a much higher systolic pulmonary arterial pressure in Gr_≥70_ than in Gr_60__–70_, suggesting the potential for the development of reactive pulmonary hypertension in Gr_≥70_. We speculated that a preoperative supra-normal LVEF may reflect the condition in which the left ventricle maximally compensates for volume overload. Supra-normal LVEF may be a surrogate for a greater MR burden, rather than being the generally known concept of LVEF.

With respect to long-term changes, post-LVEF reached a plateau of approximately 60% in patients with preoperative LVEF ≥ 50%. Notably, the mortality rate was lower in Gr_60–70_ than in Gr_50–60_. The 2017 ACC/AHA guidelines offer LVEF 60% as a cut-off for LV dysfunction, and suggest that mitral valve surgery is reasonable before LVEF reaches 60% in asymptomatic patients with chronic primary MR, with a level of evidence of B or C-LD [1]. A previous study investigated the mortality rate according to pre-LVEF (<50%, 50–60%, and ≥60%) in patients with primary MR after surgical correction and found hazard ratios 2.8 and 1.8 in patients with pre-LVE < 50% and 50–60%, respectively, compared to those of patients with pre-LVEF ≥ 60% [16]. Most previous studies referred in the guidelines adopted LVEF 60% for LV dysfunction and then compared outcomes between surgically and medically treated patients with normal pre-LVEF [17,18], or compared the recovery of post-LVEF with the normal level irrespective of the measurement time points [4,5,7,19]. Few studies have demonstrated the optimal cut-off of LVEF for predicting long-term outcomes. Meanwhile, our findings may suggest the cut-off of LVEF for defining LV dysfunction in terms of long-term outcomes after MVr, supporting the current guidelines. However, adjustment for many possible covariates is needed to determine this association.

In Gr_≥70_, the long-term post-LVEF seemed to decrease after reaching a peak. A previous study reported that pre-LVEF > 60% did not guarantee LVEF recovery during 10 years of follow-up after MVr in patients with primary chronic MR, in that LVEF returned to the preoperative level only in two thirds of patients with postoperative LV dysfunction although all patients showed pre-LVEF > 60% [7]. Moreover, a supra-normal LVEF (defined as LVEF ≥ 65%) was recently reported to show higher mortality rates than LVEF 60–65% in a large, heterogeneous clinical cohort [20]. We think that our results in Gr_≥70_ may be in line with this recent report, in that both studies suggest that supra-normal LVEF should not be considered the same as normal LVEF. Further studies are definitely needed to elucidate this issue. Various modalities for LV assessment, including cardiac magnetic resonance imaging to evaluate myocardial fibrosis [21], B-type natriuretic peptide as a biomarker for LV dysfunction, LV global longitudinal strain to assess LV function, left atrial strain as a marker of reversible cardiac dysfunction [22], and ventricular-arterial coupling as a recognized parameter of global cardiovascular performance [23], may improve the understanding of the biomechanics of LV change.

In Gr_<50_, further studies including larger numbers of such patients are necessary to identify the long-term changes in post-LVEF, because there were only 15 patients in this group.

LVESD is known to be indicative of reduced LV systolic function in patients with chronic MR [24,25,26]. A previous study demonstrated that postoperative LV dysfunction after correction of MR could be predicted with a reduced pre-LVEF and larger pre-LVESD [2]. Another study showed the additive value of pre-LVESD to pre-LVEF for predicting post-LVEF < 50% after MVr [5]. Likewise, the clinical significance of LVESD was supported by our observation that the post-LVEF was higher in patients with pre-LVESD < 40 mm than in those with pre-LVESD ≥ 40 mm with the same pre-LVEF range.

The present study had some limitations. First, this was a retrospective observational study. The measurement time points for follow-up echocardiography were determined at the physician’s discretion. Therefore, the intervals between the measurement time points differed according to individual patients and were variable even in the same patient. Some patients underwent echocardiographic examination less frequently over time after surgery, and the interpretation of these estimates of long-term results may be limited. This should be considered when interpreting the results. In addition, systolic blood pressure measurement was available in a few patients; therefore, the same was true for sc-mFS. Second, since the present study had a small number of deaths, the number of covariates included in the multivariable model was limited to avoid a potential problem of overfitting. Further studies including a large number of patients are needed to confirm independent association between pre-LVEF and long-term mortality. In addition, our result of multivariable analysis showed a wide range of 95% confidence interval that may be attributed to a small number of deaths, which should be taken into account when interpreting the result. Third, we did not have information about MR-related symptoms, symptom duration, or the time interval between the onset of symptoms and MVr, which may be related to the extent of LV remodeling. Therefore, these factors may be indicative of the LV function even in the same LVEF range. Further studies are needed to elucidate these issues.

## 5. Conclusions

In terms of short-term change, the higher the pre-LVEF was, the greater was the decrease in the immediate post-LVEF, and the immediate post-LVEF decreased more with larger pre-LVESD in the same pre-LVEF range. In terms of long-term changes, post-LVEF showed a plateau in patients with pre-LVEF > 50%, and lowest mortality was observed in patients with 60% ≤ pre-LVEF < 70%. In addition, there may also be a possibility that post-LVEF showed a decreasing trend in the long-term in patient with pre-LVEF ≥ 70%. Further studies are needed to confirm our findings.

## Figures and Tables

**Table 1:** Baseline patient characteristics and echocardiographic findings.

Variables (*n* = 461)	
Clinical data	
Age (years)	53.0 (42.0–62.5)
Male	291 (63.1)
Body mass index (kg/m^2^)	24.5 (22.4–26.5)
Diabetes mellitus	34 (7.4)
Hypertension	172 (37.3)
Atrial fibrillation	81 (17.6)
Preoperative medication	
ACEI/ARB	219 (47.5)
β blocker	101 (21.9)
Calcium channel blocker	146 (31.7)
Digoxin	71 (15.4)
Diuretics	229 (49.7)
Preoperative echocardiography	
LV ejection fraction (%)	64.6 (60.7–68.3)
LV end-diastolic volume index (mL/m^2^)	92.9 (76.3–113.6)
LV end-systolic volume index (mL/m^2^)	32.3 (26.0–40.8)
LV end-diastolic diameter (mm)	60.0 (56.0–64.0)
LV end-systolic diameter (mm)	38.0 (34.0–42.0)
Mitral regurgitation	
moderate/moderate to severe/severe	19 (4.1)/17 (3.7)/425 (92.2)
prolapse/Flail/both	302 (65.5)/152 (33.0)/7 (1.5)

Data are presented as the median (interquartile range) or number (percentage). ACEI: angiotensin converting enzyme inhibitor; ARB: angiotensin receptor antagonist; LV: left ventricular.

**Table 2:** LV volume, afterload, and systolic function.

		Gr_<50_ (*n* = 15)	Gr_50–60_ (*n* = 74)	Gr_60–70_ (*n* = 281)	Gr_≥70_ (*n* = 85)	*p*-Value ^a^	*p*-Value ^b^
LV volume (mL/m^2^)							
EDVI	Pre	116.7 ± 26.8	99.4 ± 28.4	93.0 ± 26.1 *	100.0 ± 29.8	0.002	0.188
(preload)	Post	98.2 ± 23.9	74.9 ± 20.4 *	66.3 ± 19.0 *^,^^†^	65.6 ± 20.9 *^,^^†^	<0.001	<0.001
	Diff	−18.5 ± 25.5	−24.5 ± 22.8	−26.7 ± 19.2	−34.4 ± 22.6 *^,^^†^^,‡^	0.003	<0.001
ESVI	Pre	58.2 (52.8–81.7)	39.0 (34.1–51.0) ^§^	32.0 (26.1–39.7) ^§^^,^^∥^	26.4 (21.3–31.8) ^§^^,^^∥^^¶^	<0.001	<0.001
	Post	61.4 (52.0–77.1)	34.7 (27.5–42.9) ^§^	29.0 (22.1–37.1) ^§^^,^^∥^	26.6 (20.1–35.1) ^§^^,^^∥^	<0.001	<0.001
	Diff	−11.9 (−19.2–9.3)	−5.2 (−12.8–−0.4)	−1.8 (−7.9–2.4) ^∥^	0.6 (−5.2–7.2) ^∥^^¶^	<0.001	<0.001
LV afterload (kdyne/cm^2^)							
cESS	Pre	177.1 ± 38.4	155.8 ± 43.2	140.1 ± 32.8 *	129.0 ± 35.0 *^,^^†^	<0.001	<0.001
	Post	196.5 ± 59.9	158.9 ± 57.0	135.6 ± 37.0 *	116.7 ± 27.5 *^,^^†^^,‡^	<0.001	<0.001
LV systolic function (%)							
mFS	Pre	14.1 ± 2.3	18.6 ± 2.8 *	20.1 ± 2.8 *^,^^†^	20.6 ±2.5 *^,^^†^	<0.001	<0.001
	Post	11.1 ± 2.3	14.8 ± 2.8 *	15.0 ± 2.9 *	15.4 ± 2.8 *	<0.001	<0.001

LV: left ventricular; Pre: preoperative; Post: immediately postoperative; Diff: difference between pre and post value; EDVI: end-diastolic volume index; ESVI: end-systolic volume index; cESS: circumferential end-systolic stress; mFS: midwall fractional shortening. ^a^ means *p*-value for analysis of variance (ANOVA) or Kruskal–Wallis test, as appropriate. ^b^ means *p*-value for linear trend test. In pairwise comparison after ANOVA, * *p* < 0.05 versus Gr_<50_; ^†^ *p* < 0.05versus Gr_50–60_; ^‡^ *p* < 0.05 versus Gr_60–70_. In pairwise comparison after Kruskal–Wallis test, ^§^ *p* < 0.00833 versus Gr < 50; ^∥^*p* < 0.00833 versus Gr_50–60_; *p* < 0.00833 versus Gr_60–70_; ^¶^ *p* < 0.00833 versus Gr_60–70_.

## Data Availability

The data presented in this study are available on request from the corresponding author with a reasonable reason.

## Supplementary Materials

The following are available online at https://www.mdpi.com/article/10.3390/jcm10132830/s1, Figure S1: Preoperative PG_TR_, E/e´, and LAD in patient with chronic primary mitral regurgitation, Figure S2: Restricted cubic spline curves of LVEF changes in patients in whom MR with grade ≥ moderate did not develop again during the follow-up after MVr for chronic primary MR, Table S1: Immediate postoperative LVEF after excluding patients with preoperative atrial fibrillation.
