# Supplementary material for: Changes in Left Ventricular Ejection Fraction after Mitral Valve Repair for Primary Mitral Regurgitation
Source: J Clin Med. 2021 Jun 26;10(13):2830. doi: 10.3390/jcm10132830 (PMC8267705; doi:10.3390/jcm10132830)
Supplement: Supplementary file 1 [file jcm-10-02830-s001.zip › jcm-1263734-supplementary.pdf]

Supplementary Methods

Echocardiographic data (1)

Two-dimensional echocardiography and Doppler colour flow imaging were performed in all patients using a Hewlett-Packard Sonos 2500, 5500, or 7500 imaging system (Hewlett-Packard, Andover, MA, USA) and a VIVID 7 or E9 ultrasound system (General Electric Healthcare, Little Chalfont, UK) with a 2.5 MHz probe. The left ventricular (LV) end-diastolic diameter and LV end-systolic diameter were measured from parasternal M-mode acquisitions, and the LV end-systolic volume and LV end-diastolic volume were measured using the biplane Simpson method. LV ejection fraction was calculated from the measured LV end-systolic volume and end-diastolic volume. LV end-systolic volume and end-diastolic volume index were calculated by dividing volume by body surface area. The left atrial diameter was the anteroposterior diameter measured form parasternal long-axis view. The ratio of peak early diastolic velocity of mitral inflow to mitral annulus early diastolic velocity (E/e‘) was calculated. Measurements were averaged over three to five cardiac cycles for patients with atrial fibrillation.

Comprehensive echocardiographic evaluation of mitral regurgitation was performed using an integrated approach including 2-dimensional, Doppler, and color flow imaging. The proximal isovelocity surface area (PISA) was determined by measuring the proximal flow convergence by lowering the imaging depth and reducing the Nyquist limit at mid-systole. Various views were evaluated for optimal visualization of the PISA. Baseline shift was used to adjust the aliasing velocity to about 40 cm/sec. With the simplified PISA method, the degree of MR was graded as mild (PISA radius < 4 mm), moderate (PISA radius < 8 mm), or severe (PISA radius ≥ 8 mm).

Echocardiographic data (2)

The analyzed immediate postoperative echocardiographic data were those obtained before

patient discharge. If a postoperative echocardiographic examination was performed several times before discharge because of the administration of an inotropic drug or the application of a mechanical assist device, the echocardiographic data obtained after stopping the inotropic drug or the mechanical device were used as the immediate postoperative echocardiographic data. However, if all echocardiographic examinations before discharge were performed with the administration of an inotropic drug or application of a mechanical assist device, the corresponding data were excluded in the comparison of echocardiographic data between the preoperative and immediate postoperative periods.

Echocardiographic data (3)

The actual midwall fractional shortening (mFS) was determined using the two-shell method of Shimizu et al [1]. Circumferential end-systolic stress (cESS), a measure of ventricular afterload, was calculated at the midwall according to the method of Gaasch et al [2]. Thereafter, the predicted mFS was determined for any given cESS using the regression equation derived from a healthy population [3]. To minimize afterload dependence, stress corrected mFS (sc-mFS) was calculated as the ratio of actual to predicted Mfs [3].

The preoperative sc-mFS (pre-sc-mFS) and immediate postoperative sc-mFS (post-sc-mFS) were calculated in 184 and 177 patients, respectively, because of limited data of systolic blood pressure at the time of echocardiographic examination.

References

[1] Shimizu G, Hirota Y, Kita Y, Kawamura K, Saito T, Gaasch WH. Left ventricular midwall mechanics in systemic arterial hypertension. Myocardial function is depressed in pressure-overload hypertrophy. Circulation 1991;83:1676-84.

[2] Gaasch WH, Zile MR, Hoshino PK, Apstein CS, Blaustein AS. Stress-shortening relations and myocardial blood flow in compensated and failing canine hearts with

pressure-overload hypertrophy. Circulation 1989;79:872-83.

[3] de Simone G, Devereux RB, Roman MJ, Ganau A, Saba PS, Alderman MH *et al.* Assessment of left ventricular function by the midwall fractional shortening/end-systolic stress relation in human hypertension. J Am Coll Cardiol 1994;23:1444-51.

Statistical analysis

Analysis of variance (ANOVA) was used to compare normally distributed data among the four groups, and the Tukey test for equal variance or Games-Howell test for nonequal variance was used as the post-hoc test. Kruskal-Wallis test was used to compare non-normally distributed data among the four groups, and Bonferroni correction was used as the post-hoc test (adjusted $\alpha = 0.05/6 = 0.0083$). The P for trend test using linear regression analysis or Spearman's correlation analysis was performed to investigate whether the immediate postoperative echocardiographic parameters have a linear trend across the four groups. Student's t-test or Mann-Whitney U-test was used to compare the difference between pre-LVEF and immediate post-LVEF according to pre-LVESD (≥ 40 versus < 40 mm), as appropriate. The comparisons according to pre-LVESD in the same pre-LVEF range were performed in Gr $_{\mathbf{50\text{-}60}}$, Gr $_{\mathbf{60\text{-}70}}$, and Gr $_{\mathbf{\geq 70}}$, because there was no one with pre-LVESD < 40 mm in Gr $_{\mathbf{<50}}$.

To evaluate the effect of time on post-LVEF, linear mixed models were constructed, with group, time, and the interaction between group and time as fixed effects, and patient effect as random effects. Linear function, quadratic function, and restricted cubic spline functions with the number of knots (with each 3, 4, or 5 knots) for time effects were considered. Thereafter, these five candidate functions were compared based on the Akaike information criterion to obtain the best one. Kaplan-Meier analysis was performed to compare long-term mortality using log-rank sum test, and Bonferroni correction was used as the post-hoc test (adjusted $\alpha = 0.05/6 = 0.0083$). $P < 0.05$ was considered statistically significant. We conducted all analyses

using the R version 3.5.2 packages “lme4” and “rms”.

Supplementary Results

Preoperatively, the ratio of peak early diastolic velocity of mitral inflow to mitral annulus early diastolic velocity (E/e′) did not show a significant difference among groups, and the left atrial (LA) diameter was higher in Gr $_{50\text{-}60}$ than in Gr $_{60\text{-}70}$. Moreover, the preoperative pressure gradient calculated from peak tricuspid regurgitation (PG_{TR}) was higher in Gr $_{\geq 70}$ than in Gr $_{60\text{-}70}$.

Supplementary Figures

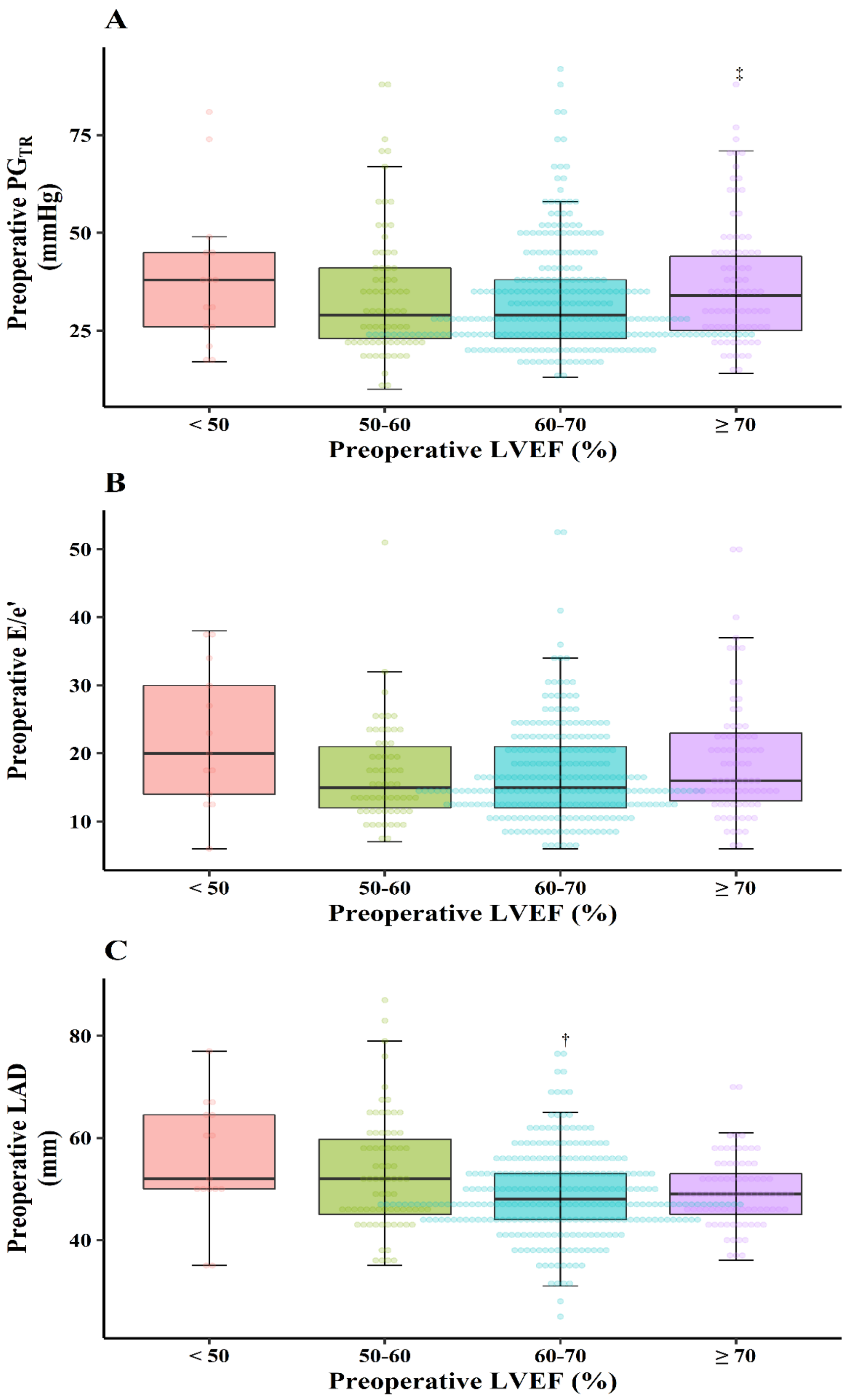

Supplementary Figure 1: Preoperative PG_{TR}, E/e′ and LAD in patient with chronic primary mitral regurgitation

Preoperative E/e′ did not show a significant difference among groups, and the preoperative PG_{TR} was higher in patients with preoperative LVEF (pre-LVEF) ≥ 70% than in those with pre-LVEF 60-70%. LVEF: left ventricular ejection fraction; PG_{TR}: pressure gradient calculated from peak tricuspid regurgitation; E/e′: ratio of peak early diastolic velocity of mitral inflow to mitral annulus early diastolic velocity; LAD: left atrial diameter.

In pairwise comparison after Kruskal-Wallis test, [‡] P < 0.00833 versus pre-LVEF 60-70.

In pairwise comparison after Analysis of variance, [†] P <0.05 versus pre-LVEF 50-60.

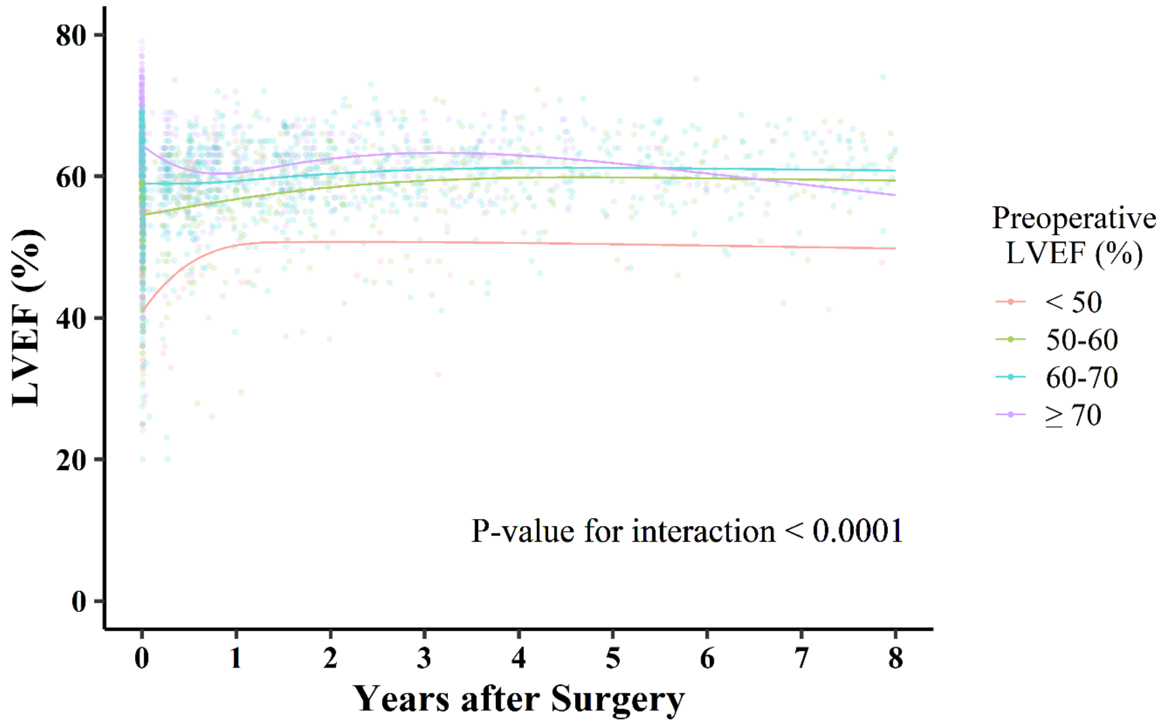

Supplementary Figure 2: Restricted cubic spline curves of LVEF changes in patients in whom MR with grade ≥ moderate did not develop again during the follow-up after MVr for chronic primary MR

The long-term postoperative LVEF (post-LVEF) reached a plateau of approximately 60% when the preoperative LVEF (pre-LVEF) was ≥ 50%, but seemed to show a downward trend after reaching a peak at approximately 3-4 years after MVr when the pre-LVEF was ≥70%. In patients with pre-LVEF < 50%, post-LVEF increased until about 1 year after MVr, and thereafter showed a plateau of approximately 50%. LVEF: left ventricular ejection fraction; MR: mitral regurgitation; MVr: mitral valve repair.

P-value was based on the interaction between the groups and the time.

Supplementary Table 1: Immediate postoperative LVEF after excluding patients with preoperative atrial fibrillation

	Gr $_{<50}$ (n=9)	Gr $_{50\text{-}60}$ (n=54)	Gr $_{60\text{-}70}$ (n=240)	Gr $_{\geq 70}$ (n=75)	*P*-value[a]	*P*-value[b]
Post-LVEF (%)	41.8 ± 7.4	51.3 ± 9.0[*]	53.7 ± 8.8[*]	57.0 ± 6.8[*†‡]	<0.001	<0.001
Difference (%)	-1.2 ± 6.9	-5.9 ± 9.0	-11.0 ± 8.7[*†]	-15.5 ± 7.3[*†‡]	<0.001	<0.001

Data are presented as mean ± SD.

LVEF: left ventricular ejection fraction; Post-LVEF: immediate postoperative LVEF; Difference: calculated as post-LVEF minus preoperative LVEF.

[a] means P-value for Analysis of variance (ANOVA)

[b] means P-value for linear trend test

In pairwise comparison after ANOVA, [*] $P < 0.05$ versus pre-LVEF < 50%, [†] $P < 0.05$ versus pre-LVEF 50-60%, [‡] $P < 0.05$ versus pre-LVEF 60-70%.
